# Postprandial apoE Isoform and Conformational Changes Associated with VLDL Lipolysis Products Modulate Monocyte Inflammation

**DOI:** 10.1371/journal.pone.0050513

**Published:** 2012-11-28

**Authors:** Laura J. den Hartigh, Robin Altman, Romobia Hutchinson, Jitka Petrlova, Madhu S. Budamagunta, Sarada D. Tetali, Jens O. Lagerstedt, John C. Voss, John C. Rutledge

**Affiliations:** 1 Division of Cardiology, Department of Internal Medicine, University of California Davis, Davis, California, United States of America; 2 Department of Biochemistry and Molecular Medicine, University of California Davis, Davis, California, United States of America; 3 Department of Experimental Medicine, Lund University, Lund, Sweden; 4 Department of Plant Sciences, University of Hyderabad, Hyderabad, India; University of Padova, Italy

## Abstract

**Objective:**

Postprandial hyperlipemia, characterized by increased circulating very low-density lipoproteins (VLDL) and circulating lipopolysaccharide (LPS), has been proposed as a mechanism of vascular injury. Our goal was to examine the interactions between postprandial lipoproteins, LPS, and apoE3 and apoE4 on monocyte activation.

**Methods and Results:**

We showed that apoE3 complexed to phospholipid vesicles attenuates LPS-induced THP-1 monocyte cytokine expression, while apoE4 increases expression. ELISA revealed that apoE3 binds to LPS with higher affinity than apoE4. Electron paramagnetic resonance (EPR) spectroscopy of site-directed spin labels placed on specific amino acids of apoE3 showed that LPS interferes with conformational changes normally associated with lipid binding. Specifically, compared to apoE4, apoE bearing the E3-like R112→Ser mutation displays increased self association when exposed to LPS, consistent with a stronger apoE3-LPS interaction. Additionally, lipolysis of fasting VLDL from normal human donors attenuated LPS-induced TNFα secretion from monocytes to a greater extent than postprandial VLDL, an effect partially reversed by blocking apoE. This effect was reproduced using fasting VLDL lipolysis products from *e3/e3* donors, but not from *e4/e4* subjects, suggesting that apoE3 on fasting VLDL prevents LPS-induced inflammation more readily than apoE4.

**Conclusion:**

Postprandial apoE isoform and conformational changes associated with VLDL dramatically modulate vascular inflammation.

## Introduction

Postprandial activation of monocytes has been implicated as a pro-inflammatory and pro-atherogenic condition [1], [2], and the repetitive injury of vascular cells during postprandial states could be an important mechanism in the development of atherosclerotic cardiovascular disease. Lipopolysaccharide (LPS), a form of endotoxin, is a principal component of gram-negative bacterial cell walls and induces a potent host inflammatory response. Recent studies have shown that consumption of a meal high in fat increases circulating endotoxin levels [3], presumably from gut flora absorbed with dietary lipids [4], [5]. Furthermore, plasma lipoproteins provide an important defense mechanism against LPS-induced inflammation by neutralizing and clearing LPS-associated lipoproteins by the liver [6].

Consumption of a meal high in saturated fat results in a transient rise in circulating triglycerides contained in chylomicrons (exogenous pathway) and very low-density lipoproteins (endogenous pathway). Circulating postprandial triglyceride peaks about 3–4 hours after the meal and usually returns to fasting levels by 6 hours post-consumption [7]. Postprandial VLDL have been implicated as atherogenic lipoproteins [8]–[10], and the effects of circulating VLDL lipolysis on vascular function have recently been under intense investigation [11]–[13]. Lipolysis of VLDL by lipoprotein lipase results in the release of smaller remnant particles and free fatty acids, monoglycerides, diglycerides, and phospholipids (defined herein as lipolysis products) in close proximity to endothelial cells and circulating monocytes [14]. While it is known that lipoproteins such as VLDL can neutralize LPS, the mechanism by which they do so remains unclear. Further, the ability of lipoproteins to neutralize LPS has not been compared between the fasted and fed states, and it is not known if lipoprotein lipolysis products influence binding and neutralization of LPS to prevent cellular injury.

ApoE exists as three major isoforms: apoE2, apoE3, and apoE4. All are exchangeable between circulating lipoproteins, most commonly associated with chylomicrons, VLDL, and high-density lipoprotein (HDL). ApoE3 is the most prevalent isoform in the general population, and differs from apoE4 by a single amino acid substitution at position 112, cysteine (apoE3) to arginine (apoE4). Approximately 15% of the population carries at least one apoE4 allele, which has been correlated with increased risk of atherosclerosis and Alzheimer’s disease [15], [16]. In addition, mechanisms by which apoE4 and apoE3 promote pro- and anti-inflammatory conditions, respectively, remain unclear [17]. ApoE4 associates primarily with VLDL in the postprandial state, and lipolysis of VLDL induces a distinct conformational change in the apoE4 protein [18]. Although it is thought that apolipoproteins such as apoE can bind to LPS [19], isoform-specific variability has not been demonstrated.

The purpose of this study was to determine if apoE isoforms differentially protect monocytes from LPS-induced activation, and how fasting and postprandial VLDL lipolysis products containing apoE3 or apoE4 influence this process. Furthermore, we investigated the hypothesis that apoE3 attenuates LPS-induced cellular activation by binding directly to LPS with a higher affinity than apoE4. We have determined that apoE3 protects THP-1 monocyte-like cells from LPS-induced inflammatory cytokine secretion, while apoE4 exacerbates monocyte activation. In addition, apoE from fasting VLDL lipolysis products is more protective against LPS-induced monocyte activation than postprandial VLDL lipolysis products. Finally, we show that the C-terminal domain of apoE3 interacts with LPS, providing a mechanism for its sequestration from monocytes.

## Methods

### Ethics Statement

Two cohorts of human subjects were recruited for blood donation. The first consisted of healthy volunteers, 18–55 years of age and representative of both genders, recruited from the University of California, Davis campus. The second included subjects of either apoE3/E3 (n = 3) or apoE4/E4 (n = 3) genotype recruited from the greater Sacramento, California area by the Alzheimer’s Disease Center of the University of California, Davis. Both studies were approved by the Human Subjects Research Committee of the University of California, Davis. The study aims and protocol were explained to each participant, and informed written consent was obtained.

### Cell Culture

THP-1 human monocytes were purchased from American Type Culture Collection and maintained in suspension between 5×10^4^ and 8×10^5^ cells/ml in RPMI 1640 medium, and used in experiments at a concentration of 1×10^6^ cells/mL. All monocyte experiments included treatment with 0.5 µg/mL LPS derived from *Escherichia coli* (Sigma-Aldrich, L2654). Monocyte viability was monitored after all treatments using trypan blue exclusion and a Live/Dead Viability/Cytotoxicity kit from Molecular Probes.

### ApoE3- and ApoE4-DMPC Preparations

Small unilamellar dimyristoyl phosphatidylcholine (DMPC, 10 mg/mL) vesicles were made by sonication in a water bath and extruded using a mini-extruder (Avanti Polar Lipids, Alabaster, AL), as described previously [20]. Recombinant apoE3 and apoE4 (purchased from EMD Biosciences) were added to DMPC vesicles at a lipid:protein ratio of 4∶1 (w/w), vortexed briefly for 10 seconds, and incubated for 1 hour at 25°C to allow protein lipidation, as described previously [21].

### Quantitative Real-Time PCR

THP-1 monocytes (1×10^6^ cells per mL) were treated with the indicated combinations of DMPC vesicles (200 µg/mL), apoE3 (50 µg/mL), apoE4 (50 µg/mL), and LPS (0.5 µg/mL) for 3 hours at 37°C. Cells were pelleted and RNA isolated using TRIzol reagent as described previously [22]. Total RNA was quantified using a Nanodrop-1000 system. Total RNA (2 µg) was used to make cDNA with a Superscript II RNase H-reverse transcriptase kit according to the manufacturer’s guidelines (Invitrogen). Quantification of mRNA from gene transcripts of tumor necrosis factor-α (TNFα), interleukin 1-β (IL-1β), and beta-actin was performed using the GeneAmp 7900 HT sequence detection system (Applied Biosciences), as described previously [23]. Primers for the genes of interest were designed using Primer Express (Applied Biosystems) and synthesized by Integrated DNA Technologies. The primer sequences were as follows: TNFα (sense, 5′- AACATCCAACCTTCCCAAACG -3′; antisense, 5′- CCCTAAGCCCCCAATTCTCTT-3′), IL-1β (sense, 5′-AATTTGAGTCTGCCCAGTTCCC-3′; antisense, 5′-AGTCAGTTATATCCTGGCCGCC-3′), beta-actin (sense, 5′- CTGTCCACCTTCCAGCAGATGT-3′; antisense, 5′- CGCAACTAAGTCATAGTCCGCC-3′).

### ApoE ELISA

To determine if apoE3 and apoE4 bind to LPS, an ELISA protocol was developed. After determining the optimal LPS coating concentration, 96-well plates were coated with LPS at 5 µg/ml and incubated at 4°C overnight for apoE capture. Unbound LPS was removed by gentle washing with 1X PBS +0.05% Tween-20. Plates were blocked for 1 hour at room temperature with 1 X PBS +10% fetal bovine serum (FBS), and recombinant apoE isoforms, with or without prior complexing to DMPC, were added at the indicated final concentrations for 2 hours at room temperature to allow binding to coated LPS. After washing away unbound apoE3 and apoE4, a monoclonal apoE antibody (EMD Millipore) was added for 1 hour at room temperature followed by secondary antibody (anti-mouse IgG-HRP, 1∶3000) for one hour. TMB substrate solution (BD Biosciences) was prepared by mixing equal volumes of reagent A and reagent B and added to each well for 30 minutes to detect the peroxide-labeled secondary antibody. The colorimetric reaction was stopped by addition of 1 M phosphoric acid, and the absorbance was read within 10 minutes. ApoE3 and apoE4 adhesion to LPS was represented as a dose-dependent increase in absorbance values at 450 nm. Control wells, with and without LPS coating, containing either apoE alone, apoE+DMPC, apoE vehicle, apoE antibody, anti-mouse antibody, or substrate solution contained no measureable absorbance (data not shown), indicating specific LPS-apoE interactions were measured. DMPC alone background controls did not exceed absorbance levels above the lowest apoE concentrations used.

### ApoE Mutant Cloning and Purification

The gene encoding human apoE4 protein containing the W264→Cys (apoE4-W264C) substitution was cloned into the commercial vector pET151/D-Topo (Invitrogen) according to the manufacturer’s instructions. The N-terminal fusion tag was removed by introducing flanking BamHI sites by PCR mutagenesis, followed by BamHI digestion and then ligation, so that the first residue of apoE (Lys) in the expressed protein is preceded by the amino acid sequence M-G-S. To generate the apo E3-like protein containing the same Cys substitution at position 264, the R112→S mutation in the apoE4-W264C template was introduced by PCR mutagenesis. Fidelity of cloning and mutations was confirmed by DNA sequencing.

For protein expression, apoE4-W264C and apoE3-like-W264C were transformed into BL21(DE3)AI *Escherichia coli* cells (Invitrogen). Cells were grown in LB broth (1 L) to mid-log phase at 37°, then induced with 0.2% arabinose followed by incubation for 3 hours at 37°. Cells were harvested by centrifugation, and inclusion bodies isolated and washed as described previously [24]. To purify the protein, washed inclusion bodies were dissolved in 8 M urea, and the sample filtered through a 0.2 micron filter and then separated on a SuperDex size exclusion column containing 100 µM tris-(2-carboxyethyl)phosphine (TCEP) to maintain reduced disulfides. The fractions compiling the predominant protein peak were pooled, and the protein spin labeled with 0.4 mM MTS-SL (Toronto Research Chemicals) for 30 minutes. Denatured samples were scanned and double-integrated to determine labeling efficiency, which measures at least 95% at position 264 under the TCEP labeling conditions. The protein was re-folded and separated from free spin- label using an ion exchange column (GE Health Sciences) connected to a Pharmacia FPLC chromatography system. The pool of labeled apoE protein was loaded onto the column, washed, and then eluted using a NaCl gradient. The major eluted peaks were pooled, concentrated using spin concentrators (Millipore), and the protein concentration was determined using the Pierce BCA kit (Thermo Scientific).

### Electron Paramagnetic Resonance (EPR) Spectroscopy

EPR measurements were carried out in a JEOL TE-100 X-band spectrometer fitted with a loop-gap resonator as described previously [24]. LPS was added to the nitroxy spin-labeled protein (4 mg/mL) at a final concentration of 30 µg/mL for 2 hours prior to EPR measurements. Appropriate vehicle controls were used for all samples. Approximately 5 µL of the protein was loaded into a sealed quartz capillary tube. The spectra were obtained by averaging two 2-minute scans with a sweep width of 100 G at a microwave power of 4 mW and modulation amplitude optimized to the natural line width of the attached spin probe. All the spectra were recorded at room temperature.

### Isolation of Very Low-Density Lipoproteins (VLDL) and Remnants

All studies were performed at the same time of day to eliminate any diurnal variables. Following a 12-hour fast, subjects were fed a moderately high fat (40% calories from fat) meal as described previously [7]. Blood was drawn by venipuncture from subjects pre- and post-prandially (3.5 hours following meal consumption) into K_2_-EDTA vacutainer tubes and centrifuged at 1,200 g for 10 minutes to obtain cell-free plasma. Plasma was treated with 0.01% sodium azide as a preservative and subjected to lipoprotein isolation as described previously [25], with minor modifications. Chylomicrons were removed from postprandial plasma by centrifuging for 30 minutes at 63,000 g prior to VLDL isolation. Lipid samples were dialyzed overnight at 4°C in 0.9% NaCl and 0.01% EDTA and quantified using an autoanalyzer for total triglyceride and apoE content. Purity of lipid fractions was visualized by TITAN lipoprotein gel electrophoresis (gels obtained from Helena Laboratories). Lipolysis of VLDL was allowed by addition of lipoprotein lipase (LpL, 2 U/mL, obtained from Sigma-Aldrich, St. Louis, MO) for 30 minutes at 37°C prior to treatment of monocytes. Remnants were further isolated by density gradient ultracentrifugation of hydrolyzed VLDL, and characterized for total triglyceride, apoE, and apoB content using a PolyChem Analyzer using manufacturer’s reagents (MedTest DX, Cortlandt Manor, NY).

### Tumor Necrosis Factor-α ELISA

THP-1 monocytes were treated with VLDL, hydrolyzed VLDL, or VLDL remnants for 3 hours. All treatments included LPS at 0.5 µg/mL. A monoclonal apoE antibody (EMD Biosciences) was used for blocking experiments by incubating it with hydrolyzed VLDL at 4°C for 15 minutes at a final dilution of 1∶1000. Total secreted TNFα protein was quantified using an ELISA kit from BD Biosciences according to the manufacturer’s instructions, and normalized to the LPS control(s).

### Statistics

All statistical analyses were performed using SigmaStat software. All results are reported as mean ± SEM, as indicated. A student t-test was used to elucidate significant differences between apoE3 and apoE4 binding affinity to LPS, with group comparisons determined significant for P<0.01, as indicated. One-way ANOVA was used to determine significance for all other experiments, with pairwise comparisons performed using the Holm-Sidak method. Statistical significance was reported for P<0.05 or P<0.01, as indicated.

## Results

### ApoE3 and apoE4 Differentially Alter Monocyte Gene Expression in Response to LPS

**Figure 1:**
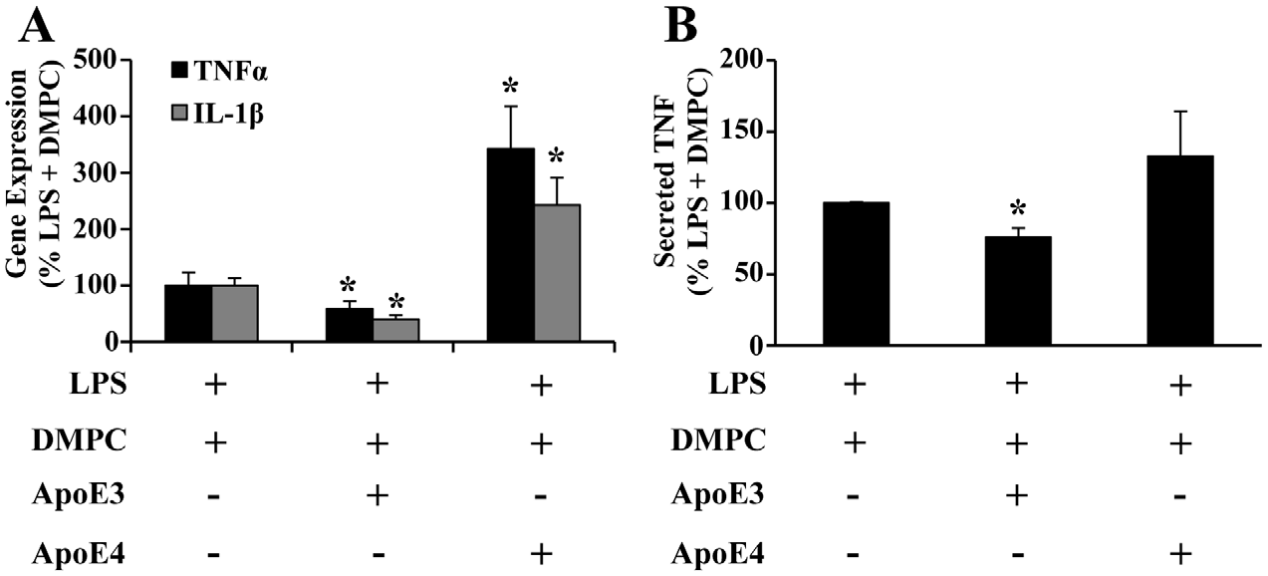
Differential effects of apoE3 and apoE4 on LPS-induced cytokine expression and secretion. ApoE3 or apoE4 were complexed with extruded DMPC vesicles, then LPS was added for treatment of THP-1 cells. (A) TNFα and IL-1β gene expression and (B) TNFα protein secretion were quantified and normalized to the LPS+DMPC control for each treatment, (n = 5). *P<0.05.

To determine if apoE3 and apoE4 influence LPS-induced cytokine expression, THP-1 cells were treated with recombinant apoE3 and apoE4 complexed with DMPC. As shown in Figure 1A, ApoE3-DMPC attenuated LPS-induced TNFα and IL-1β gene expression to 58% and 40% of the LPS+DMPC control, respectively. Conversely, apoE4-DMPC increased TNFα and IL-1β gene expression to 340% and 240%, respectively. There was no significant effect on expression of either gene upon treatment with DMPC alone, apoE vehicle, DMPC+apoE vehicle, or either apoE isoform without LPS (not shown). Secreted TNFα showed a similar trend towards an attenuated response with ApoE3-DMPC and an enhanced response with ApoE4-DMPC (Figure 1B). These results suggest that apoE3 is protective against LPS-induced cellular injury, while apoE4 potentiates it, which is consistent with our previous studies in human endothelial cells [21].

### ApoE3 Binds to LPS with Greater Affinity than apoE4

**Figure 2:**
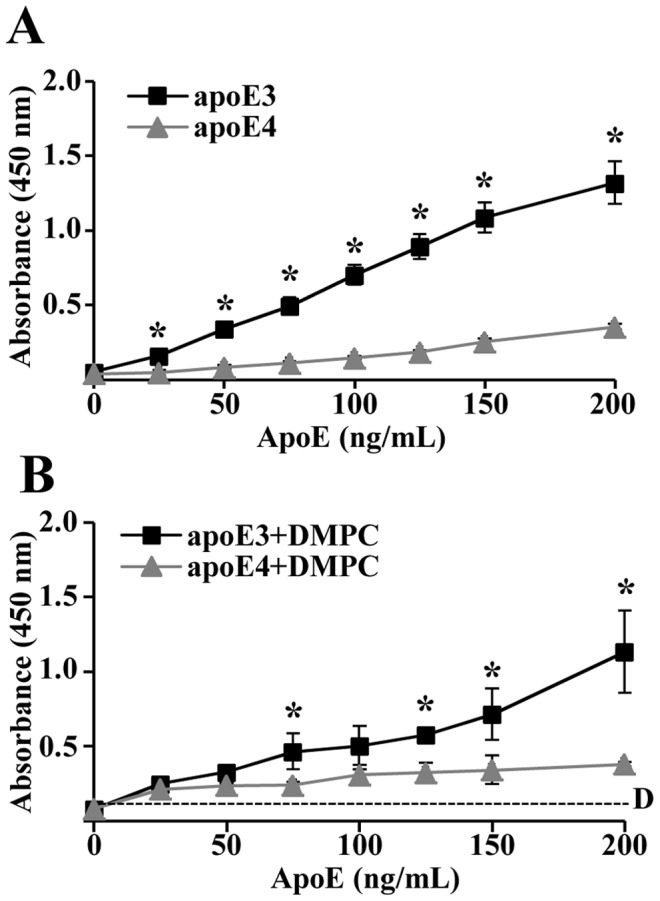
ApoE3 binds LPS with greater affinity than apoE4. LPS-coated wells were incubated with increasing concentrations of apoE3 or apoE4 alone (A) or complexed to DMPC (B). LPS-bound apoE was detected using an apoE antibody with secondary HRP, with adhesion directly proportional to absorbance (n = 6). Dashed line, DMPC background. *P<0.01.

To determine if apoE3 and/or apoE4 directly bind to LPS, an apoE ELISA was developed. Human recombinant apoE3 and apoE4 were titrated in equal concentrations between 0 and 1000 ng/mL in separate wells of an LPS-coated ELISA plate. Binding is represented as the absorbance output values at 450 nm. ApoE3 bound LPS more avidly than apoE4 in the linear range of the titration, between 0 and 200 ng/mL (Figure 2A). ApoE3 complexed to DMPC resulted in similar levels of LPS binding (Figure 2B), suggesting that LPS also interacts with lipid-bound apoE3. Appropriate ELISA controls, such as no LPS, resulted in no measureable absorbance detected. These data indicate that both lipid-free and lipid-bound apoE3 has higher affinity for LPS than apoE4.

### ApoE3 Exhibits Greater C-terminal Domain Interactions than apoE4 when Associated with LPS

The C-terminal region of apoE has been identified as the protein’s principal lipid binding domain [26], with this distinction attributed to a stronger interaction between amino acids in the N- and C-terminal domain of apoE4 [20]. As shown by Tetali et al. [18], a decrease in the self-association of lipid-free apoE4 protein is observed via the decreased broadening of the EPR spectrum resulting from a diminished magnetic coupling between spin labels attached to position Trp264 in the C-terminal domain of the protein [27]. Upon lipid binding, the self-association of the C-terminal domain is disrupted, resulting in larger EPR amplitude due to the decreased dipolar broadening. We therefore investigated the effect of LPS on the lipid-induced structural conversion of apoE4. Since apoE3 contains a Cys at position 112, specific spin labeling at position 264 is not possible. However, previous work has shown that the Arg→Ser substitution at position 112 imparts E3-like behavior to apoE4 [28]. We constructed the mutant apoE3-like W264C+Arg112→Ser to evaluate whether the LPS binding differences observed between the isoforms by ELISA are reflected by a differential conformation of the apoE C-terminal domain.

**Figure 3:**
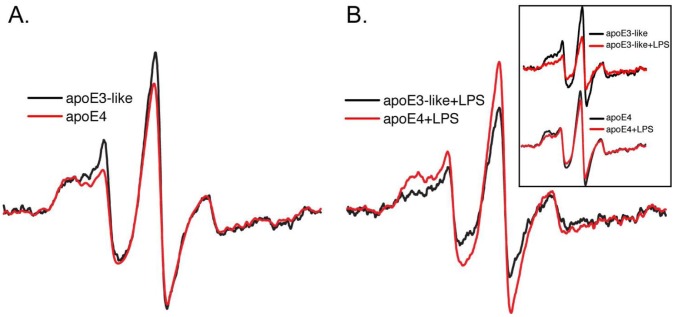
Interaction of apoE C-terminal domain is increased by LPS. EPR spectra of spin-labeled apoE4-264C and E3-like apoE-W264C+Arg112→Ser in the absence (A) and presence (B) of LPS. LPS induces spectral broadening of both apoE4 and the E3-like proteins (inset), but the magnitude of the effect is greater with E3-like protein (B).

As shown in Figure 3, spin labeled apoE4-264C and the apoE3-like W264+Arg112→Ser proteins were examined by EPR spectroscopy in the absence and presence of 30 µg/mL LPS. Figure 3A shows the spectra of each protein (normalized to the same number of spins) in the absence of LPS. Consistent with greater domain interaction in the E4 isoform, the spectrum of the spin-label attached to apoE4-264C is slightly broader compared to its attachment at the same location in the E3-like protein [29]. The spectra are nearly identical for each apoE mutant protein. Figure 3B compares the same protein samples after treatment with LPS. Remarkably, in contrast to lipids in the form of VLDL or synthetic emulsions [18], both proteins display increased C-terminal interaction in the presence of LPS (apparent by the increased spectral broadening, see inset). Consistent with the higher affinity of apoE3 for LPS, the spectral effect with the E3-like protein is substantially larger. This indicates that the spectral difference observed with LPS treatment is significantly higher for the E3-like protein than it is for apoE4. When spin labels were placed on sites in the N-terminal apoE4 domain (position 57, 76, or 77), no LPS-dependent changes are observed (data not shown).

### Fasting VLDL Lipolysis Products Attenuate LPS-induced Monocyte TNFα Secretion in a Dose-dependent Manner

**Figure 4:**
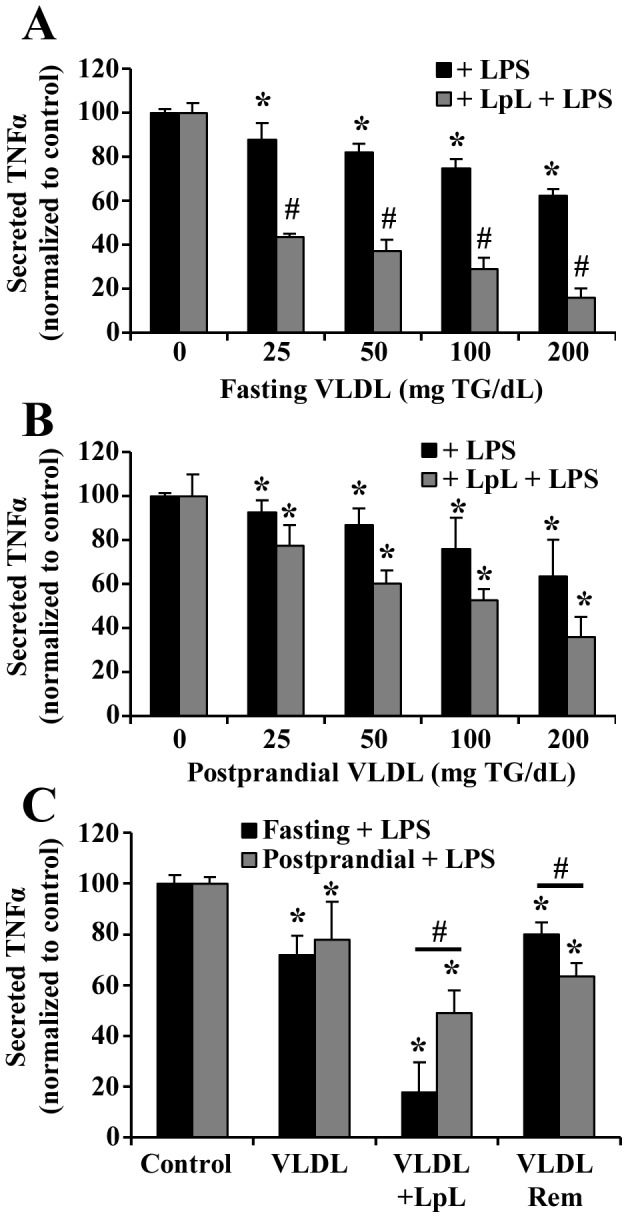
Fasting VLDL lipolysis products attenuate LPS-induced TNFα release. (A-B) Monocytes treated with fasting or postprandial VLDL lipolysis products and LPS exhibited dose-dependent attenuation of TNFα secretion (n = 6). A: fasting VLDL; B: postprandial VLDL. *P<0.01 from control, #P<0.05 from the same concentration dose without LpL. (C) Postprandial remnants (VLDL rem) attenuate LPS-induced TNFα secretion more than fasting remnants. *P<0.05 from control, #P<0.05 from fasting.

We have previously shown that the postprandial state induces structural changes in apoE isoforms, with a minor role attributed to triglyceride-rich lipoprotein lipolysis products [18]. In the circulation, VLDL particles become hydrolyzed by lipoprotein lipase (LpL) into smaller remnant particles and fatty acids, monoglycerides, diglycerides, and phospholipids. Additionally, lipoproteins such as VLDL have been shown to interact with circulating LPS by an unknown mechanism [19], [30]. To determine the effects of these lipolysis products on monocyte activation by LPS, lipolysis of fasting and postprandial VLDL from normal donors over a range of triglyceride (TG) concentrations was performed by addition of LpL (2 U/mL) for 30 minutes at 37°C. LPS (0.5 µg/mL) was added to each VLDL or VLDL+LpL sample, and was immediately incubated with THP-1 monocytes for 3 hours. A dose-dependent decrease in TNFα secretion was observed from monocytes treated with fasting VLDL+LPS (Figure 4A, black bars), and also from monocytes treated with postprandial VLDL+LPS (Figure 4B, black bars). However, lipolysis of fasting VLDL significantly enhanced the attenuation from LPS stimulation beyond that for fasting VLDL (Figure 4A, grey bars), while lipolysis of postprandial VLDL showed no statistically significant difference from postprandial VLDL attenuation (Figure 4B, grey bars). A VLDL dose of 200 mg TG/dL was chosen for subsequent experiments based on the differences in attenuation between fasting and postprandial VLDL lipolysis products at this dose.

VLDL lipolysis products attenuated the LPS-induced inflammatory response more than VLDL, despite the abundance of fatty acids released upon lipolysis, some of which may be pro-inflammatory. To determine if the remnant lipoproteins were capable of attenuating LPS-induced TNFα secretion more than VLDL or VLDL lipolysis products, remnants were isolated after the lipolysis reaction and used for monocyte treatment. Figure 4C shows that remnants from both fasting and postprandial VLDL were no more protective than VLDL, and less protective than all lipolysis products, suggesting that lipolysis of fasting VLDL renders apoE more capable of sequestering LPS. In addition, postprandial VLDL remnants were more protective than fasting VLDL remnants.

To ensure that the different effects of VLDL lipolysis products from the fasting and postprandial state were not due to variations in apolipoprotein or LPS content, total apoE protein, apoB protein, and endotoxin were measured and normalized to triglyceride content (Table 1). Using an autoanalyzer from Polymedco, we found that the fasting and postprandial VLDL lipolysis samples had an average total apoE content of 3.4±0.5 and 3.5±0.6 mg apoE/g TG, respectively. After isolating the VLDL remnants, apoE content dropped to 0.53±0.23 and 0.84±0.32 mg apoE/g TG for fasting and postprandial samples, respectively. The endotoxin levels in all VLDL samples averaged 0.25±0.2 endotoxin units per mL, which is regarded as below the acceptable endotoxin levels for cell cultures, using a Limulus Amebocyte Lysate (LAL) kit from Cambrex.

**Table 1:** ApoE and apoB content of VLDL lipolysis products and remnants (n = 3).

	Fasting samples		Postprandial samples	
	ApoE content mg/g TG(% VLDL)	ApoB content mg/g TG(% VLDL)	ApoE content mg/g TG(% VLDL)	ApoB content mg/g TG(% VLDL)
VLDL+LpL	3.4±0.50(122%)	16.0±3.0(101%)	3.5±0.60(99.2%)	15.3±1.7(107%)
VLDL remnants	0.53±0.23(12.2%)	2.7±0.33(22.9%)	0.84±0.32(12.1%)	2.3±0.37(15.9%)

### Blocking apoE Associated with Fasting VLDL Lipolysis Products Partially Reversed the Attenuation of LPS-induced TNFα Release from Monocytes

**Figure 5:**
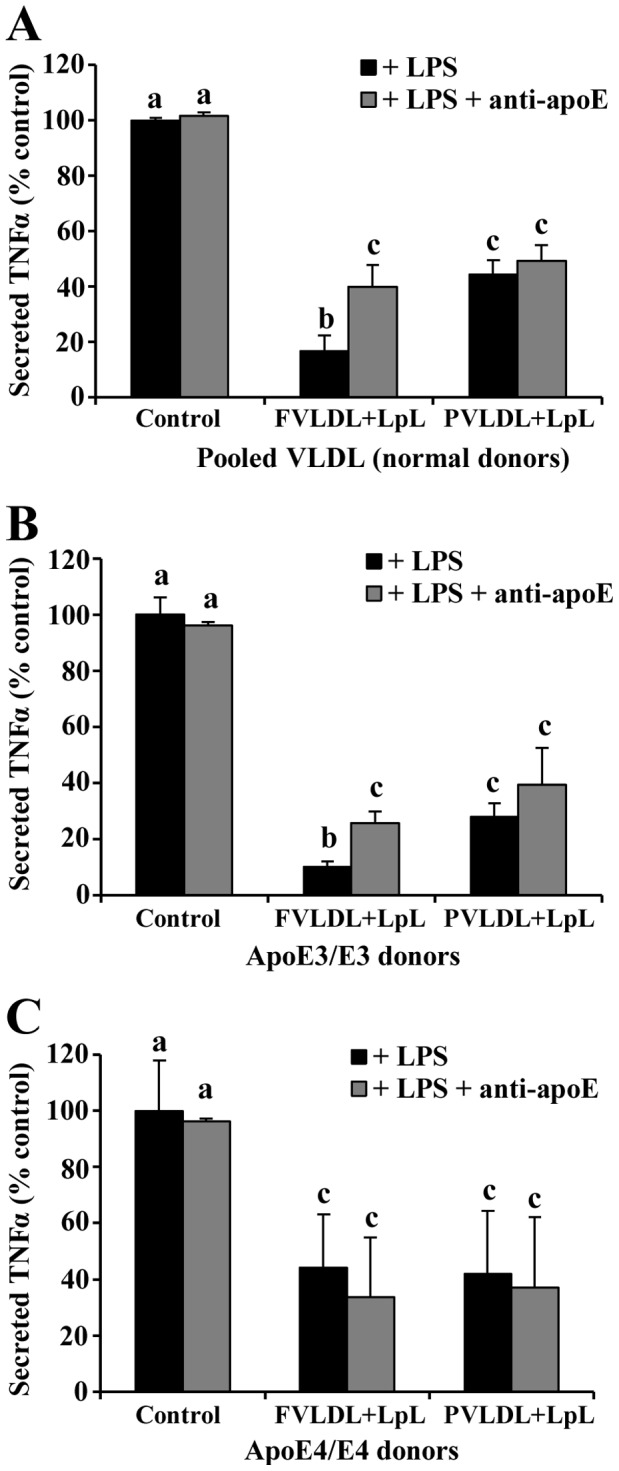
Blocking apoE from fasting VLDL lipolysis products reverses the attenuation of LPS-induced TNFα release. Monocytes were treated with apoE-blocked VLDL lipolysis products from (A) pooled normal (n = 6), (B) apoE3/E3 subjects (n = 3), and (C) apoE4/E4 subjects (n = 3). Blocking fasting samples reversed the attenuated TNFα release, except for apoE4/E4. Different letters represent significant differences between groups; P<0.01 for a vs. b and a vs. c; P<0.05 for b vs. c.

To determine if apoE present on hydrolyzed fasting or postprandial VLDL is involved in the attenuation of LPS-induced monocyte activation, apoE was blocked prior to addition of LPS using a monoclonal apoE antibody. Blocking apoE on fasting VLDL lipolysis products pooled from normal donors reversed the attenuation of the LPS-induced TNFα secretion from monocytes from 17% to 53% of the LPS control (Figure 5A). Blocking apoE associated with lipolysis of postprandial VLDL did not significantly change the level of TNFα secreted (44% to 49%), implying that apoE associated with fasting VLDL lipolysis products has a greater potential to reduce LPS-induced inflammation than apoE on postprandial VLDL.

We further evaluated the protective effects of native apoE3 and apoE4 on VLDL obtained from healthy donors with apoE3/E3 and apoE4/E4 genotypes. As shown in Figure 5B, the monocyte responses to fasting and postprandial VLDL lipolysis products containing apoE3 were similar to those from normal pooled subjects containing unknown (but presumably apoE3) apoE isoforms. Blocking apoE on apoE3-containing VLDL partially reversed the attenuation of TNFα secretion. However, the reduced inflammatory response to fasting and postprandial apoE4-containing VLDL lipolysis products was less striking (Figure 5C). Furthermore, blocking apoE on apoE4-VLDL lipolysis products had no effect on TNFα secretion levels, suggesting that apoE4 is not involved in the protective effect of apoE4-containing VLDL lipolysis products.

**Figure 6:**
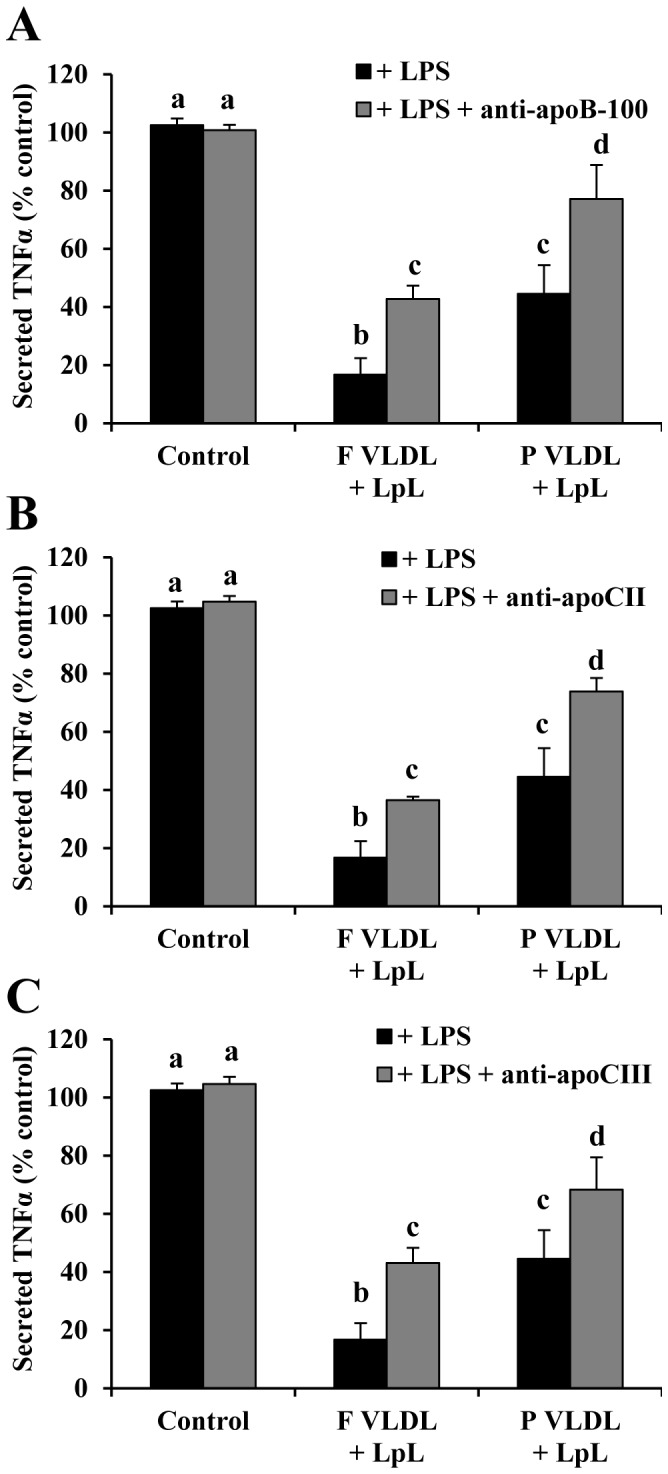
Blocking apoB-100, apoCII, and apoCIII on VLDL lipolysis products reverses the attenuation of LPS-induced TNFα secretion. Monocytes were treated with (A) apoB-100, (B) apoCII, or (C) apoCIII-blocked fasting and postprandial VLDL lipolysis products from pooled normal subjects (n = 3). Blocking each apolipoprotein from both fasting and postprandial samples reversed the attenuated TNFα release. Different letters represent significant differences between groups; P<0.01 for a vs. b and c; P<0.05 for a vs. d.

Since blocking apoE only partially reversed the attenuated TNFα secretion, the contributions of other apolipoproteins present on VLDL were examined. Using monoclonal antibodies, we blocked apoB-100, apoCII, and apoCIII from pooled VLDL samples (Figure 6). Blocking all of these apolipoproteins resulted in a reversal of the attenuation of the LPS inflammatory response, similar to blocking apoE. However, the effect was the same when blocking the apolipoproteins on fasting VLDL lipolysis products as on postprandial VLDL lipolysis products. Therefore, no differential effect was seen between fasting and postprandial samples after blocking apoB-100, apoCII, or apoCIII. This implies that apoE, but not the other apolipoproteins, undergoes a functional change in the postprandial period.

## Discussion

Triglyceride-rich lipoproteins are known to bind and neutralize LPS, thereby preventing sepsis-induced host death [31], [32]. It has been suggested that apolipoproteins, and in particular apoE, play a role in reducing LPS-induced lethality [19]. The distribution and structural conformation of apoE on lipoproteins undergo dramatic changes during the postprandial period, which may alter its ability to interact with LPS. The objective of this study was to compare the effects of apoE3 and apoE4 on LPS-induced activation of monocytes.

A differential effect of apoE isoforms on endotoxemic inflammation has been suggested by previous studies. Microglial cells from mice homozygous for the apoE4 allele display pro-inflammatory characteristics including increased cytokine expression and altered cell morphology [33], while transgenic mice expressing apoE4 have an increased NFκB-dependent pro-inflammatory expression profile [34], [35]. Conversely, macrophages expressing apoE3 are less responsive to LPS than those expressing apoE2 or apoE4 [36], presumably by reducing TLR4-mediated signaling through JNK [37]. Zhu et al. have suggested that apoE3 attenuates LPS-induced cytokine expression from these macrophages by enhancing cholesterol clearance, thus rearranging plasma membrane cholesterol domains necessary for adequate TLR4 signaling [37]. Using a synthetic phospholipid emulsion, we have shown that apoE3 reduces LPS-induced monocyte activation, while apoE4 potentiates it. A similar observation has been made previously in human aortic endothelial cells, in which the inflammatory response to TNFα was blunted by apoE3 and augmented by apoE4 [21]. It is apparent that there are several potential mechanisms involving both direct and indirect LPS action by which apoE3 and apoE4 exert opposing effects on the inflammatory response, presumably by differentially regulating NFκB-mediated cell signaling, and which warrant further studies.

In contrast to apoE3, apoE4 induced an inflammatory response from monocytes when treated in combination with LPS. We investigated whether the binding kinetics between apoE isoforms and LPS were different. Using an ELISA that we developed, there was significantly more direct binding of LPS to apoE3 than to apoE4 within the linear range of the dose-response (between 0 and 200 ng/mL). This suggests that LPS directly interacts with apoE3 preferentially over apoE4, which could explain the protection from LPS-induced inflammation associated with apoE3. However, the lower binding capacity of apoE4 to LPS does not explain the augmented inflammatory response above that for the LPS+DMPC control, which may be due to additional interactions between apoE4 and specific cellular signaling pathways.

Previous EPR studies have demonstrated that apoE self-associates via its C-terminal domain [38]. LPS induces substantial broadening of the EPR spectrum from labels attached at the C-terminal domain of the apoE3-like protein. A similar, but smaller, spectral change of apoE4 occurs when incubated with LPS. This suggests that LPS stabilizes the interaction between C-termini, to a greater extent in the apoE3-like mutant than apoE4. Examination of three different sites in the N-terminal domain did not reflect any conformational changes, indicating the C-terminus primarily binds to LPS. Thus position 264 serves as an especially useful marker for studying the influences of LPS on apoE. Given that the C-terminal domain represents the region of the protein with the highest lipid-binding affinity, factors that stabilize protein-protein interactions may alter the distribution of apoE on lipoprotein particles, and as such have profound effects on lipid metabolism or downstream processes linked to inflammation [39].

After consumption of a high fat meal, VLDL released from the liver undergoes lipolysis in close proximity to the endothelial surface of the vascular wall, exposing passing monocytes to high concentrations of lipolysis products. Here we report a protective effect of hydrolyzed VLDL on subsequent monocyte activation by LPS, with protection being greater when the lipolysis substrate is fasting VLDL rather than postprandial VLDL. There was no significant difference in secreted TNFα attenuation between fasting and postprandial VLDL treatment without lipolysis. Our observation that LPS interacts with the C-terminal lipid binding domain of apoE, combined with the enhanced protection provided by hydrolyzed apoE-containing VLDL against LPS, suggests that lipolysis of VLDL exposes the C-terminal domain of apoE, which could explain the added protection provided by VLDL lipolysis products compared with whole VLDL. Furthermore, apoE3 was found to be involved in this protective effect associated with fasting VLDL lipolysis products, but not involved in protecting monocytes from LPS when associated with postprandial VLDL lipolysis products. The total apoE content of both fasting and postprandial VLDL used in each experiment was similar, and therefore any variations in apoE-LPS interactions could be attributed to changes in isoform or conformation. Our data suggest that apoE becomes more available for LPS neutralization when VLDL is hydrolyzed, particularly from fasting VLDL. In contrast, the remnant particles isolated following hydrolysis of postprandial VLDL were more protective against LPS-induced inflammation than fasting remnants, supported by higher apoE content. Our previous studies also have shown that apoE4 preferentially associates with VLDL while apoE3 associates with HDL in postprandial plasma [18]. This change in apoE distribution could explain the reduced capacity for postprandial VLDL lipolysis products to attenuate LPS-induced inflammation [18], [40]. The conformation of apoE, and therefore its affinity for LPS, could become altered postprandially, which could account for the reduction in postprandial protection from LPS-induced injury.

Several explanations could account for the difference between fasting and postprandial VLDL and protection from LPS-induced monocyte inflammation. First, our previous study showed that lipoprotein lipase itself could exert pro- or anti-inflammatory effects on endothelial cells depending on the specific stimulating agent [41]. However, this seems unlikely in this case given that LpL alone did not attenuate LPS-induced inflammation. Second, lipolysis products from VLDL, such as free fatty acids, could counteract the actions of LPS. We have recently shown that lipolysis of postprandial VLDL generates approximately two-fold more non-esterified fatty acids than lipolysis of fasting VLDL [42], and that these fatty acids alone induce pro-inflammatory gene expression from THP-1 monocytes [43]. Third, VLDL lipolysis products may differ in their composition when released from fasting or postprandial VLDL, yielding a net greater anti-inflammatory effect when monocytes are treated with fasting VLDL lipolysis products and LPS. Using laser trapping Raman spectroscopy, we previously showed that individual fasting VLDL are rich in unsaturated lipid modes, while postprandial VLDL contain highly ordered saturated modes [44]. This compositional difference between fasting and postprandial VLDL suggests that lipolysis of fasting VLDL would release more unsaturated fatty acids than postprandial VLDL. Other studies have shown that treatment of monocytes with polyunsaturated fatty acids (PUFA) decreases cellular activation with LPS co-stimulation [45], while saturated fatty acids have been implicated as pro-inflammatory [46], suggesting that the composition of the VLDL lipolysis products could contribute to an overall pro-inflammatory or anti-inflammatory response. We have recently characterized postprandial VLDL lipolysis products from VLDL samples pooled from normal human subjects [47]. As expected, these contain high levels of saturated (348.48 nmol/mg TG), monounsaturated (227.55 nmol/mg TG), and polyunsaturated (204.8 nmol/mg TG) non-esterified fatty acids, of which C16∶0, C18∶1n9/1n7t, and C18∶2n6 were the most abundant. With more unsaturated fatty acids released per mg triglyceride, it seems plausible that these could contribute to inflammatory protection by activating anti-inflammatory and LPS-counteracting signaling pathways such as those controlled by PPARγ.

In conclusion, apoE3 prevents LPS-induced inflammatory responses from monocytes, while apoE4 exaggerates them. We have shown for the first time that LPS binds directly to apoE3, which could explain some of the anti-inflammatory properties associated with apoE3. Further, apoE3 plays a role in blunting the inflammatory response to LPS activation, especially when associated with hydrolyzed fasting VLDL. Our findings provide insights into complex postprandial lipoprotein metabolism experienced multiple times per day in persons consuming the typical Western diet. This knowledge of the pro- and anti-inflammatory actions of apoE and VLDL and their lipolysis products will facilitate the development of therapeutic strategies to prevent and attenuate atherosclerotic cardiovascular disease.
